# Substrate utilization during submaximal exercise in children with a severely obese parent

**DOI:** 10.1186/1743-7075-9-38

**Published:** 2012-05-09

**Authors:** Audrey D Eaves, Ashley Colon, Katrina D DuBose, David Collier, Joseph A Houmard

**Affiliations:** 1 Department of Kinesiology, College of Health and Human Performance, East Carolina University, Greenville, USA; 2 Human Performance Laboratory, College of Health and Human Performance, East Carolina University, Greenville, USA; 3 Department of Pediatrics, Brody School of Medicine, East Carolina University, Greenville, USA; 4 East Carolina Diabetes and Obesity Institute, East Carolina University, Greenville, USA; 5 Human Performance Laboratory, Ward Sports Medicine Building, East Carolina University, Greenville, NC, 27858, USA

**Keywords:** Bariatric surgery, Class III obesity, Exercise, Fat oxidation, Skeletal muscle

## Abstract

**Background:**

We have reported a reduction in fatty acid oxidation (FAO) at the whole-body level and in skeletal muscle in severely obese (BMI ≥ 40 kg/m^2^) individuals; this defect is retained in cell culture suggesting an inherent component. The purpose of the current study was to determine if an impairment in whole-body fatty acid oxidation (FAO) was also evident in children with a severely obese parent.

**Methods:**

Substrate utilization during submaximal exercise (cycle ergometer) was determined in children ages 8–12 y with a severely obese parent (OP, n = 13) or two lean/non-obese (BMI range of 18 to 28 kg/m^2^) parents (LP, n = 13). A subgroup of subjects (n = 3/group) performed 4 weeks of exercise training with substrate utilization measured after the intervention.

**Results:**

The children did not differ in age (LP vs. OP, respectively) (10.7 ± 0.5 vs. 10.2 ± 0.5 y), BMI percentile (65.3 ± 5.2 vs. 75.9 ± 7), Tanner Stage (1.4 ± 0.2 vs. 1.5 ± 0.2), VO_2_peak (40.3 ± 2.7 vs. 35.6 ± 2.6 ml/kg/min) or physical activity levels (accelerometer). At the same absolute workload of 15 W (~38% VO_2_peak), RER was significantly (*P* ≤ 0.05) lower in LP vs. OP (0.83 ± 0.02 vs. 0.87 ± 0.01) which was reflected in a reduced reliance on FAO for energy production in the OP group (58.6 ± 5.1 vs. 43.1 ± 4.0% of energy needs during exercise from FAO). At a higher exercise intensity (~65% VO_2_peak) there were no differences in substrate utilization between LP and OP. After exercise training RER tended to decrease (*P* = 0.06) at the 15 W workload, suggesting an increased reliance on FAO regardless of group.

**Conclusions:**

These findings suggest that the decrement in FAO with severe obesity has an inherent component that may be overcome with exercise training.

## Background

In the United States, the incidence of severe or class III obesity (BMI ≥ 40 kg/m^2^) is increasing at a rate 2 to 3 times faster than lower range obesity [1]. The severely obese condition has a profound effect on health care; while a BMI of 35 to 40 kg/m^2^ was associated with a 50% increase in health care expenditures, a BMI of ≥ 40 kg/m^2^ doubled health care costs above those of normal weight [2]. In relation to the underlying etiology of this disease, our research group has consistently found that fatty acid oxidation (FAO) is impaired at the whole body level [3,4] and specifically in skeletal muscle [5-7] with severe obesity. This decrement in FAO may contribute to the development of the severely obese state, as it has been reported that individuals who exhibit a lower rate of fat oxidation are prone to weight gain [8]. The impairment in FAO is still evident after the pronounced weight loss induced by gastric bypass surgery [3-5] and remains intact in muscle cell cultures raised from severely obese donors [9,10] both of which suggest a resilient trait. Exercise training, however, rescued FAO in previously severely obese individuals who exhibited this initial deficit [5].

It is difficult to determine whether the depressed FAO seen with severe obesity is a cause or a consequence of the disease. Parental obesity increases the risk of becoming an obese adult by more than two-fold regardless whether the child is obese or nonobese [11]. It has also been reported that normal weight subjects with a family history of obesity display reduced lipid oxidation implying a genetic contribution [12]. However, FAO in the offspring of specifically severely obese individuals has, to our knowledge, not been examined. The purpose of the present study was to determine if the reduction in FAO seen in adults with severe obesity was also evident in children with at least one severely obese parent and if exercise training could correct this condition. We have previously been able to detect the reduction in FAO with severe obesity using a submaximal exercise protocol [4]; we thus utilized this non-invasive approach to examine whole-body FAO in children.

## Methods

### Experimental design and research subjects

The purpose of this study was to compare substrate utilization during submaximal exercise in prepubescent children (8 to 12 y) with at least one severely obese (BMI ≥ 40 kg/m^2^) parent versus children with two non-obese parents (BMI ≤ 28.0 kg/m^2^). A subgroup of children from each group was also examined after a 4-week endurance-oriented exercise program to determine if substrate utilization could be altered.

Children with a severely obese parent (OP) were recruited by contacting individuals who were either contemplating or had recently undergone gastric bypass or lap banding surgery to treat severe obesity. As the majority of patients undergoing weight loss surgery in our clinics are women, all of the mothers of the children were severely obese. The children with a severely obese mother who had undergone surgery were born > 5 years prior to the procedure. Medical records were checked to ensure that the parent exhibited class III obesity prior to the surgery. Children with lean/non-obese parents (LP) were recruited by flyers, newspaper advertisements, and by contacting individuals who had participated in previous studies. To be placed in the LP group, both biological parents had to possess a BMI that placed them in the normal or non-obese category (BMI between 18.5 and 28.0 kg/m^2^), been stable at this BMI for ≥ 1 y, and could not have been previously severely obese. The children in each group were matched for age, gender, and race. Children were excluded if they were on any type of medication or had a medical condition that altered metabolism, limited their ability to exercise, or posed a risk for exercise. Children were also excluded if they regularly participated in an organized sport or physical activity. All procedures were approved by the University and Medical Center Institutional Review Board at East Carolina University. Written informed consent was obtained from the parent and either written or verbal assent from the child.

### Subject characteristics

Initial screening involved at least one biological parent and the child. Questionnaires to determine Tanner stage for the child [13] and both the child and parents’ physical activity patterns [14] were completed by a parent. Height and weight were measured; if only one parent was present, height and weight were obtained for the other via self-report. Minimum waist circumference was measured in the children, and seated and standing height used to determine peak height velocity as an index of biological maturity [15,16]. A DEXA scan determined body composition. During the initial visit the child briefly rode the cycle ergometer wearing the head gear, mouth piece, and nose clips used for the maximal and submaximal exercise tests. The child was given the option of wearing an ActiGraph GT1M accelerometer (ActiGraph, Pensacola FL, USA) for one week to provide information on physical activity [17]. Instructions were given about wearing the accelerometer as well as a log sheet to record when the child put on and took off the instrument.

### Maximal exercise test

A maximal exercise test was done on a Lode Corval cycle ergometer (Lode BV, Groningen, The Netherlands) and maximal oxygen consumption assessed using indirect calorimetry (ParvoMedics True Max 2400 Metabolic cart, Sandy, UT, USA). The maximal exercise protocol was based on work by Arngrimsson, Sveinsson, and Johannsson [18] which examined 9 and 15 year old adolescents. Initial workload and the stepwise increments were 20 W if body mass was less than or equal to 30 kg or 25 W if body mass was above 30 kg; workload increased every third minute until voluntary exhaustion or a pedal rate of 60 rpm could not be maintained. Criteria for a valid test included achieving a heart rate ≥ 195 bpm, RER ≥ 1.0, or a plateau in VO_2_ despite increasing workload [18]. A second maximal exercise test was performed ≥ 4 days after the initial test.

### Submaximal exercise

Instructions for the submaximal exercise test stressed the importance of arriving in the laboratory in the morning after an overnight fast. The submaximal exercise protocol was based upon a previous study in our laboratory examining substrate utilization with severe obesity [4]. Children exercised for 10 min at identical absolute (15 W) and relative (~65% VO_2_peak) workloads in order to account for possible differences in cardiovascular fitness. The workloads were selected as they could be relatively easily maintained and relied on a mixture of substrates for energy production [4]. The order of the workloads was counterbalanced and the child given a 10 to 15 minute rest between each. The average of the final three minutes of submaximal exercise was used to determine substrate utilization [4].

### Exercise training

A subgroup of children performed 4 weeks of supervised physical activity using a program similar to that utilized by Duncan and Howley which increased fat oxidation during moderate intensity exercise [19]. Training was performed 3 days/wk at ~65% of VO_2_peak for 30 min/session during the first week which progressed to 60 min/session in week 2. Each session began with a warm-up of stretching and walking for 10 min. All training was supervised and consisted of walking on a treadmill, stationary cycling, and recreational activities such as soccer, racquetball, and tag. Participants exercised in groups to provide a social atmosphere. After the 4 weeks of training participants underwent another maximal and submaximal exercise test and height, weight, and minimal waist circumference measured.

### Statistical analyses

Data were compared between LP and OP and before and after training with a between groups and repeated measures ANOVA, respectively. Significant differences were accepted at *P* ≤ 0.05. Values are expressed as mean ± the standard error (SE).

## Results

### Parental characteristics

The majority of patients undergoing bariatric surgery are women [20]. It was thus not surprising that the severely obese parents who volunteered were women; their characteristics were compared with the mothers in the lean/non-obese parents group and are presented in Table 1. There were no significant differences between the mothers in age or stature while the severely obese women weighed more and had a higher BMI. Body mass prior to the surgery was used for the mothers in the OP group if applicable. In the fathers, there were no differences in age (38.3 ± 1.8 vs. 39.8 ± 2.1 y) or BMI (26.5 ± 1.1 vs. 30.7 ± 1.8 kg/m^2^ for LP and OP, respectively).

**Table 1:** Parental Characteristics

**Variable**	**Lean Parent**	**Obese Parent**	***P* Value**
Age, yr	39.8 ± 1.6	37.2 ±1.4	0.25
Mass, kg	68.6 ± 2.6	126.3 ±5.1*	<0.001
Height, cm	165.5 ± 1.4	168.6 ± 2.1	0.22
BMI, kg/m^2^	25.0 ± 0.8	45.4 ± 1.2*	<0.001

Characteristics of the mothers of the participants (n = 13 per group). Pre-bariatric surgery characteristics were used in the obese parents when applicable. Mean ± SE.

*P ≤ 0.05 for a difference between groups.

### Subject characteristics

Subjects were matched for age, gender and race; each group consisted of eight Caucasian boys, one Caucasian girl, two African American boys, and two African American girls. As presented in Table 2, there were no differences in age or anthropometric characteristics between the OP and LP groups. Data analyses were also performed when children ≥ 95^th^ percentile for BMI, which is the designation for obesity in children [21], (1 child from LP, 3 from OP) were excluded (BMI Z score, 0.358 ± 0.140 vs. 0.616 ± 0.241; BMI percentile, 62.8 ± 4.9 vs. 69 ± 7.8 for LP and OP, respectively) to more closely match the groups in terms of obesity status. This manipulation did not alter interpretation of the findings.

**Table 2:** Descriptive characteristics of the children

**Variable**	**LP (n = 13)**	**OP (n = 13)**	***P* Value**
Age, yr	10.7 ± 0.5	10.2 ± 0.5	0.51
Mass, kg	39.1 ± 2.4	51.3 ± 6.7	0.10
Height, cm	146.9 ± 2.8	151.8 ± 4.7	0.29
BMI, kg/m2	18.2 ± 0.6	21.3 ± 1.7	0.10
BMI Z Score	0.455 ± 0.161	1.031 ± 0.287	0.09
BMI Percentile	65.3 ± 5.2	75.9 ± 7.0	0.23
%BF DEXA	21.7 ± 2.2	26.6 ± 3.2	0.15
Minimum Waist, cm	53.5 ± 4.9	60.8 ± 7.2	0.41
Tanner Stage	1.4 ± 0.2	1.5 ± 0.2	0.77
PHV, yr	3.2 ± 0.4	3.4 ± 0.4	0.73

LP, children with lean/non-obese parents; OP, children with a severely obese parent; % BF, percent body fat determined with DEXA; PHV, calculated peak height velocity. Mean ± SE. There were no statistically significant differences between the groups.

### Maximal exercise

Each participant performed two maximal exercise tests. Comparison revealed significantly higher parameters on test two; mean data for the second test are presented in Table 3. There were no differences between the groups.

**Table 3:** Maximal exercise characteristics of the children

**Variable**	**LP**	**OP**	***P* Value**
VO_2_peak, ml/kg/min	40.3 ± 2.7	35.6 ± 2.6	0.22
VO_2_peak, L/min	1.5 ± 0.1	1.7 ± 0.2	0.46
Peak Watts	113.1 ± 10.0	118.0 ± 17.0	0.81

Characteristics obtained during a maximal exercise test on a cycle ergometer

LP, children with lean/non-obese parents; OP, children with a severely obese parent (n = 13 per group). Mean ± SE. There were no statistically significant differences between the groups.

### Submaximal exercise

**Figure 1:**
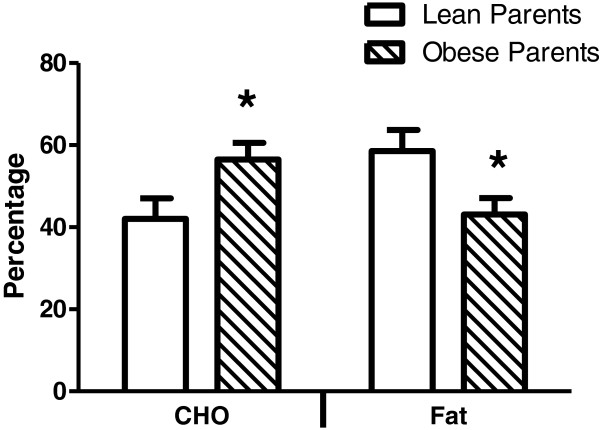
**Substrate utilization expressed as the percentage of fat or carbohydrate (CHO) contributing to total energy needs during cycling exercise at a workload of 15 watts in children with lean/non-obese parents (n = 13) or at least one severely obese parent (n = 13).** *Significantly different from children with lean/non-obese parents (*P* ≤ 0.05) for that substrate. Mean ± SE.

As presented in Table 4, oxygen consumption at an absolute workload of 15 W was not significantly different between groups when expressed in absolute or relative terms or as a percentage of VO_2_peak. There was, however, a significant difference in RER with the OP group being significantly higher than the LP group (Table 4). This reduced reliance on fat oxidation to meet the energy demands of exercise in the OP group is depicted in Figure 1. The reduced reliance on fat oxidation in OP persisted when subjects ≥ 95^th^ percentile were excluded (RER, 0.82 + 0.02 vs. 0.87 ± 0.02; % CHO, 41.2 ± 5.3 vs. 58.6 ± 5.0; % Fat, 59.6 ± 5.5 vs. 41.1 ± 4.9 for LP vs. OP respectively, *P* ≤ 0.05 for all comparisons).

**Table 4:** Responses to a submaximal exercise workload (15 W)

**Variable**	**LP**	**OP**	***P*Value**
VO_2_, ml/kg/min	14.5 ± 0.7	13.2 ± 1.2	0.19
VO_2_, L/min	0.55 ± 0.02	0.62 ± 0.05	0.35
% peak VO_2_, ml/kg/min	37.4 ± 2.4	38.0 ± 2.7	0.83
% peak VO_2_, L/min	37.5 ± 2.4	38.1 ± 2.7	0.89
Heart Rate (beats/min)	113.3 ± 4.3	109.1 ± 3.7	0.62
RER	0.83 ± 0.02	0.87 ± 0.01*	<0.05

LP, children with lean/non-obese parents; OP, children with a severely obese parent (n = 13 per group). Mean ± SE.

*P ≤ 0.05 for a difference between the groups.

As presented in Table 5 there were no significant differences between groups for many of the variables measured at a workload approximating 65% VO_2_peak. Heart rate was significantly elevated in LP vs. OP. Interpretation was not altered when obese children were excluded.

**Table 5:** **Responses to a submaximal exercise workload (65% VO**_**2**_**Peak)**

**Variable**	**LP**	**OP**	***P*Value**
VO_2_, ml/kg/min	25.5 ± 1.8	21.5 ± 1.8	0.13
VO_2_, L/min	0.98 ± 0.08	1.1 ± 0.14	0.67
% peak VO_2_, ml/kg/min	63.3 ± 2.1	60.0 ± 2.5	0.32
% peak VO_2_, L/min	63.5 ± 2.2	60.7 ± 2.4	0.39
Heart Rate (beats/min)	153.3 ± 4.3	143.8 ± 3.6*	≤0.05
RER	0.90 ± 0.01	0.91 ± 0.01	0.53

LP, children with lean/non-obese parents; OP, children with a severely obese parent (n = 13 per group). Mean ± SE. *P ≤ 0.05 for a difference between groups.

### Activity measures

Accelerometer data was obtained in 8 subjects in LP and 6 subjects in OP. Minutes per day of sedentary, light, moderate, vigorous, and total active time are reported in Table 6 and there were no significant differences between groups. Average counts per minute also did not differ between groups (LP, 794.6 ± 35.4; OP, 852.5 ± 66.3).

**Table 6:** Minutes of activity per day

**Mins./Day**	**LP (n=8)**	**OP (n=6)**
Sedentary	1023±29.30	1022±45.11
Light	325.3±18.79	331.1±39.23
Moderate	39.59±8.156	41.02±7.683
Vigorous	2.304±0.7779	4.725±1.513
Total Physical Activity	367.2±24.06	376.9±44.69

LP, children with lean/non-obese parents; OP, children with a severely obese parent. Minutes per day (min/d) at the respective activity levels were determined with an accelerometer. There were no differences between groups. Mean ± SE.

### Exercise training

**Figure 2:**
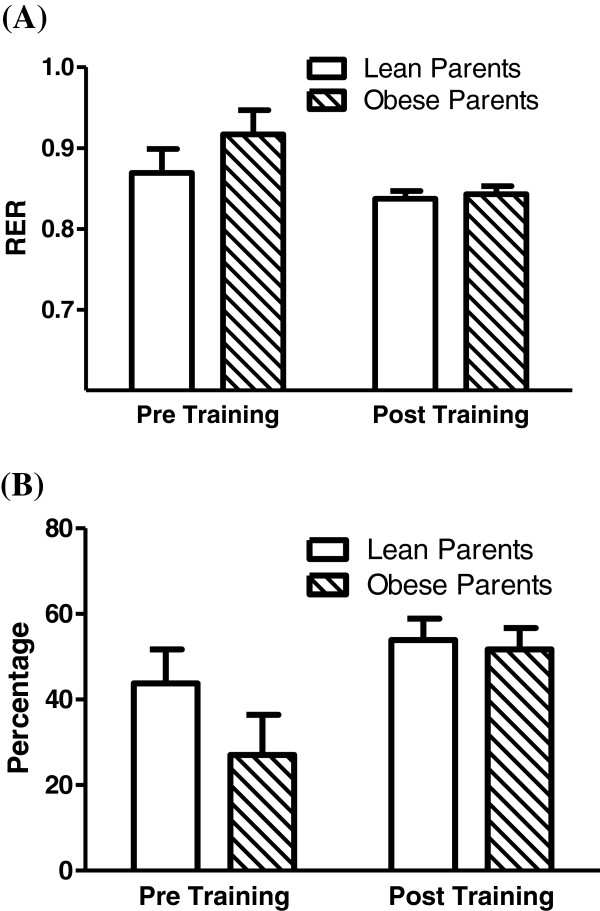
**Respiratory exchange ratio (RER) (Panel A) and the percentage of fat utilization contributing to total energy needs (Panel B) during cycling exercise at a workload of 15 W before (Pre Training) and after (Post Training) a 4 week endurance-oriented exercise training program in children with lean/non-obese parents (n = 3) or at least one severely obese parent (n = 3).** RER tended to decrease (*P* = 0.06) and fat utilization tended to increase (*P* = 0.07) with exercise training regardless of group. Mean ± SE.

Three children from each group elected to perform 4 weeks of exercise training. Each group consisted of one Caucasian female, one Caucasian male, and one African American female with no significant differences in age (10.0 ± 0.6 vs. 9.7 ± 0.9 y), mass (35.9 ± 2.5 vs. 35.7 ± 1.8 kg), BMI Z score (0.25 ± 0.23 vs. 0.28 ± 0.65), BMI percentile (60.3 ± 9.5 vs. 55.7 ± 20.3), body fat percentage (26.1 ± 3.3 vs. 23.1 ± 2.7%), Tanner Stage (1.3 ± 0.3 vs. 1.3 ± 0.3), and VO_2_peak (34.4 ± 4.5 vs. 38.1 ± 1.5 ml/kg/min; 1.2 ± 0.2 vs. 1.4 ± 0.1 l/min) (LP vs. OP, respectively) between the groups. None of these measures changed with exercise training. As presented in Figure 2, during exercise at the 15 W workload RER tended to decrease (*P* = 0.06) with exercise training which was reflected by a trend to increase the relative percentage of fat oxidation contributing to the total energy demand during exercise (*P* = 0.07). RER (mean of 0.90 across all conditions and groups) and substrate utilization at the 65% VO_2_peak workload did not differ between groups nor changed with exercise training.

## Discussion

Exposure to an obesogenic environment (i.e. high food availability, low physical activity) is a critical component contributing to positive energy balance and the development of obesity. However, genetic susceptibility can also play a role as shown by heritability estimates ranging from 40 to 70% for the obese state [22]. An experimental approach used to examine the role of genetics is to study subjects with a familial history of obesity. Using this model, Giacco et al.[12] reported that the lean offspring of overweight parents responded to a high fat meal with a significantly lower incremental increase in fat oxidation compared to offspring from normal weight parents. This impairment in the ability to effectively utilize lipid under conditions eliciting fatty acid oxidation may be a critical component of obesity, as a lower rate of lipid oxidation has been linked to weight gain in both the Pima Indians [8] and the Baltimore Longitudinal Study on Aging [23].

Our research group has reported a decrement in FAO at the whole-body level [3,4] and in skeletal muscle [7,24] with severe obesity which may predispose these individuals to weigh gain and the severely obese state. The main finding of the present study was that relatively lean children with a severely obese parent (OP) also displayed an impaired rate of FAO during mild physical activity which required predominantly lipid as the source for energy production (Figure 1, Table 3). This difference did not appear to be due to a higher level of obesity in the OP vs. LP group (Table 2, Results) nor differences in cardiovascular fitness (Table 3) and physical activity patterns (Table 6).

Using an identical exercise protocol, we previously reported that the rate of FAO during submaximal exercise at workloads eliciting 15 W and 65% VO_2_peak was reduced in sedentary, severely obese women who lost weight via gastric bypass surgery compared to sedentary, weight-matched controls who were never severely obese [4]. In the present study in children the impairment in FAO was only evident at the milder, 15 W workload (Figure 1; Tables 4,5) which offers several hypothetical scenarios concerning severe obesity. First, early in life there may be an underlying deficit in the ability to produce energy from lipid that is evident only during conditions which elicit a relatively lower degree of energy demand and thus higher proportion of FAO (i.e. the 15 W workload) (Figure 1, Table 4) which subsequently progresses into a more encompassing condition in adulthood [4]. This progression of the impairment in FAO could involve an underling genetic/epigenetic component which increases its expression and/or environmental influences that accumulate during the development of the severely obese state. Secondly, the children in the present study appeared to spend a substantial amount of time per day performing relatively mild-intensity activities (Table 6). A lower rate of FAO during such activities (Figure 1) could potentially place children with a severely obese parent in a condition of positive lipid balance compared to their counterparts and predispose them to ectopic lipid accumulation. Both of these hypotheses, however, remain to be verified.

Studies examining children with obese parents exhibit equivocal results with some indicating a low capacity for oxidative metabolism [25] while others report no relationship between indices of FAO and a predisposition to obesity [26]. Part of this discrepancy may be related to the conditions under which FAO was assessed; in the present study we utilized submaximal exercise inasmuch as deficits in the rate-limiting steps of oxidative pathways in skeletal muscle may be potentially easier to detect during conditions of elevated energy demand. Also, in our previous work we only observed a reduction in FAO in the skeletal muscle of severely obese individuals (BMI ≥ 40 kg/m^2^) and not with lower grade obesity [5,6]. The degree of obesity of the parents may thus also be a critical factor when examining metabolic characteristics that can predispose children towards weight gain. For example, a propensity for developing severe obesity may involve an epigenetic/genetic component such as a reduction in the capacity for lipid oxidation; with lower-grade obesity the epigenetic/genetic influences may not be present and thus limit the amount of body mass gain.

Although sample size was small, a relatively short course of physical activity/exercise (30–60 min/d, 3 d/wk, 4 wks) still tended to increase the contribution of FAO to total energy needs during submaximal exercise at the 15 W workload in both the LP and OP groups (Figure 2). A similar exercise protocol decreased RER and increased the rate of fat oxidation during submaximal cycling exercise at workloads ranging from approximately 30 to 70% VO_2_peak [19]. In this study by Duncan and Howley [19] children trained entirely on a cycle ergometer for 30 min/d, 3 d/wk for 4 weeks as opposed to the more general exercise program utilized in the current study (Methods). This lack of training specificity may be responsible for the absence of change at our higher (~65% VO_2_peak) workload compared to their findings [19]. However, we attempted to design an exercise program that was enjoyable and potentially clinically relevant for children; the finding of a possible enhancement in FAO thus offers promise for this intervention. In support of the efficacy of exercise, we have reported that FAO in skeletal muscle increased in previously severely obese individuals after only 10 days of endurance-oriented training [5].

The current study design cannot exclude environmental factors as a possible influence upon FAO. Dietary intake was also not controlled prior to the submaximal testing, although all subjects were examined in the fasted condition. In addition, we cannot discern possible biological mechanisms explaining the reduction in FAO nor the genes involved, both of which are outside the scope of this work. We have, however, reported that a lower capacity for lipid oxidation is retained in muscle cell cultures raised from obese donors which implies a genetic/epigenetic origin [9,10,27]; this premise is supported by the current data indicating a decrement in FAO in children with a severely obese parent.

## Conclusions

Children with a severely obese (BMI ≥ 40 kg/m^2^) parent exhibited a significantly higher RER and a reduced reliance on lipid oxidation during submaximal exercise at a mild workload (15 W or ~38% VO_2_peak) compared to children with two non-obese parents. This finding is supportive of earlier data suggestive of a genetic or epigenetic component for the reduction in FAO in adults with severe obesity [3,9,10]. With a relatively short course of endurance-oriented exercise training (4 weeks) FAO tended to increase at the 15 W workload, indicating the potential effectiveness of exercise for prevention/intervention.

## Competing interests

The authors declare they have no competing interests.

## Authors’ contributions

AE recruited subjects, obtained data, analyzed data, and participated in the drafting of the manuscript. AC recruited subjects, obtained data, analyzed data, and participated in the drafting of the manuscript. KD participated in conceptually designing the studies, interpreting the data, and drafting the manuscript. DC participated in conceptually designing the studies, interpreting the data, and drafting the manuscript. JH participated in conceptually designing the studies, statistically analyzing the data, interpreting the data, and drafting the manuscript. All authors read and approved the final manuscript.
